# Use of microwave ablation for thermal treatment of solid tumors with different shapes and sizes—A computational approach

**DOI:** 10.1371/journal.pone.0233219

**Published:** 2020-06-15

**Authors:** Masoud H. H. Tehrani, M. Soltani, Farshad Moradi Kashkooli, Kaamran Raahemifar

**Affiliations:** 1 Department of Mechanical Engineering, K. N. Toosi University of Technology, Tehran, Iran; 2 Advanced Bioengineering Initiative Center, Computational Medicine Center, K. N. Toosi University of Technology, Tehran, Iran; 3 Department of Electrical and Computer Engineering, University of Waterloo, Waterloo, ON, Canada; 4 Centre for Biotechnology and Bioengineering (CBB), University of Waterloo, Waterloo, ON, Canada; 5 Cancer Biology Research Center, Cancer Institute of Iran, Tehran University of Medical Sciences, Tehran, Iran; 6 Electrical and Computer Engineering Department, Sultan Qaboos University, Muscat, Sultanate of Oman; 7 Chemical Engineering Department, University of Waterloo, Waterloo, ON, Canada; 8 College of Information Sciences and Technology (IST), Data Science and Artificial Intelligence Program, Penn State University, State College, Pennsylvania, PA, United States of America; McGill University, CANADA

## Abstract

Microwave Ablation (MWA) is one of the most recent developments in the field of thermal therapy. This approach is an effective method for thermal tumor ablation by increasing the temperature above the normal physiological threshold to kill cancer cells with minimum side effects to surrounding organs due to rapid heat dispersive tissues. In the present study, the effects of the shape and size of the tumor on MWA are investigated. To obtain the temperature gradient, coupled bio-heat and electromagnetic equations are solved using a three-dimensional finite element method (FEM). To extract cellular response at different temperatures and times, the three-state mathematical model was employed to achieve the ablation zone size. Results show that treatment of larger tumors is more difficult than that of smaller ones. By doubling the diameter of the tumor, the percentage of dead cancer cells is reduced by 20%. For a spherical tumor smaller than 2 cm, applying 50 W input power compared to 25 W has no significant effects on treatment efficiency and only increases the risk of damage to adjacent tissues. However, for tumors larger than 2 cm, it can increase the ablation zone up to 21%. In the spherical and oblate tumors, the mean percentage of dead cells at 6 GHz is nearly 30% higher than that at 2.45GHz, but for prolate tumors, treatment efficacy is reduced by 10% at a higher frequency. Moreover, the distance between two slots in the coaxial double slot antenna is modified based on the best treatment of prolate tumors. The findings of this study can be used to choose the optimum frequency and the best antenna design according to the shape and size of the tumor.

## Introduction

Hyperthermia is one of the tumor treatment methods in which devices deliver energy to increase the temperature of the cells in body tissue. The early clinical usages were primarily for the treatment of patients who were not surgical resection candidates owing to prohibitive tumor location, multi-nodular tumors, medical comorbidities, or anatomic constraints limiting resection [1–3]. Most of the patients are not surgical resection candidates because of limitations including multifocal disease, tumor position to key vessels, and size of tumor [4]. Increasing the temperature causes the killing of cancer cells and damages the proteins and structures within the cells. MWA has great and promising potentialities for the treatment of primary and secondary liver disease and tumors in lung, kidney, and bone [5]. The use of MWA has been expanded due to many benefits when compared to other existing methods, including higher temperature, larger ablation zone when using high power, faster ablation time, decreased susceptibility to vessels in proximity to the tumor, and insensitivity to tissue features [5–7].

The purpose of thermal ablation is to heat the targeted tissue to the threshold temperature, which can induce immediate coagulative necrosis, without causing collateral damage in normal tissue [8]. The treatment efficacy is, therefore related to the ablated margins. Ablation zone profile is a function of many factors, including frequency, antenna design, ablation time, and input power [9–11]. Most of the MWA systems used a frequency spectrum of either 915 MHz or 2450 MHz. The 2450 MHz generators cause higher temperatures and shorter lesions compared to 915 MHz [12–14]. Yoon et al. [15] determined 18 GHz as an optimal frequency in the range of 0.9–30 GHz with low input power (1–3 W) to achieve MWA with lower side effects and higher efficiency to treat the small-sized tumor. Jones et al. [16] employed a new high-frequency microwave applicator with mid-range power in *ex vivo* human hepatic parenchyma and colorectal liver metastases to further study the relationship between time, power, and ablation size. Hines-Peralta [17] investigate the effect of power (50–150 W) and treatment duration in *ex vivo* liver model with 2.45 GHz microwave to achieve a larger ablation zone. They found the largest coagulated area in the maximum power and time.

The coaxial-slot antenna is very important for MWA treatment due to its low cost, simplicity, small dimensions, and high efficiency [18, 19]. Ibitoy et al. [20] analyzed the efficiency of different antennas proposed for MWA. Their results show that the sleeve antenna produces the highest sphericity temperature distribution, and a double slot antenna has the largest ablation zone and aspect ratio. Etoz and Brace [21] designed dual-slot antennas and optimized the distance between the slots for obtaining the spherical SAR pattern and also the minimum reflection. Results show 19 mm and 8 mm for the minimum reflection and spherical SAR, respectively. On the other hand, Kampatzakis et al. [22] have found a slot-to-slot distance of 10 mm to produce large, uniform, and localized lesion margins.

Temperature increase above 42–43°C for moderate periods of time causes protein denaturation and cellular functions damaged or inactivated [23, 24]. Therefore, the amount of damage is related to the achieved temperature and also duration of the treatment. Since the maximum temperature alone does not depict the ablation zone exactly [25], mathematical models are presented for cells response to temperature increase. Arrhenius models are widely employed to analyze higher temperature thermal treatments [26]. Also, they have been successfully used to predict irreversible thermal changes in structural proteins. Liljemalm and Nyberg [27] used the Arrhenius model for the cell injury to study the laser-generated heat gradients. This method has previously been used for the non-isothermal method to evaluate thermal injury behavior [28]. Huang et al. [29] used the Arrhenius model to evaluate cell death induced by extracellular hyperthermia, in which gold nanorods were irradiated with a near-infrared laser. Their results agreed well with experimental measurements. Several other studies have utilized the Arrhenius model to analyze cells response to the heat [26, 29, 30]. Such models are facing two major limitations. The first limitation is that the two governing parameters are very sensitive to experimental values. The second one is that cell survival rates for various temperatures demonstrate a certain discontinuity at temperatures around 43°C [31, 32]. O’Neill et al. [33] proposed a mathematical model of cell death for hyperthermia. They positioned the vulnerable state between living and dead states and achieved excellent correspondence with the experimental data. Hall et al. [32] performed a sensitivity analysis to determine the effects of the most important parameters of the model, i.e. blood perfusion, electrical conductivity, and the cell death model, in the prediction of RFA zone size in the liver. Lin et al. [34] utilized the three-state cell death model to predict the optimum temperature and exposure time that generates the maximum heat shock protein and reduces metastasis in nontherapeutic cancer immunotherapy by using hyperthermia.

According to the above-mentioned literature review and to the best knowledge of the authors, in none of the previous studies, the effect of tumor size and shape have ever been considered in the optimization of the effective parameters on the ablation zone. On the other hand, previous studies have shown that the antenna structure dictates the shape and size of the ablation zone, but the effect of this issue on tumor shape and size has not been considered yet [35]. For the first time, in the present study, the effect of tumor shape and size is studied on the MWA treatment process using the three-state cell death mathematical model to optimize the power and frequency according to the dimensions of the tumor. Moreover, the distance between slots in the double-slot antenna, which designed for more thermal focus in previous studies [35], is modified for a more beneficial treatment in the prolate shape tumors.

## Methods

This section includes the theory and mathematical approach employed for developing the presented model, which can be roughly divided into three parts: an electromagnetic model, a biological heat transfer model, and a cell death model. The proposed division in this section is used to simplify the understanding of theories that support the presented model. Also, model geometry, numerical approach, mesh independency, and validation of the results have been investigated and discussed.

### Electromagnetic model

A three-dimensional FEM is used to solve the electric fields and temperature profiles. The model assumes that the wall of a coaxial antenna is a perfect electric conductor (PEC). Antenna and tissue are considered as a uniform, linear, and isotropic medium. The wave equation is obtained from the Maxwell equation as [26, 36]:

$$\nabla \times \left(\nabla \times \vec{E}\right)-\mu_{r}k_{p}{}^{2}\left(\varepsilon_{r}-\frac{j\sigma }{\omega \varepsilon_{0}}\right)\vec{E}=0$$ (1)

in which $\vec{E}$, *μ*_*r*_ *k*_p_, and *ε*_*r*_ are the electric field (V/m), the differential permeability (H/m), the constant of propagation in free space (m^-1^), and the tissue relative permittivity, respectively. Also, *ε*_*0*_ = 8.854×10^−12^ F/m is the vacuum relative permittivity, *σ* is the tissue conductivity, and *ω* = 2π*f* is the angular frequency. The magnetic field can be obtained from the Maxwell-Ampere's law.

The dielectric properties of the tissue are temperature dependent [37]. The electrical conductivity (S/m) and electrical permittivity (1) is defined by a linear function of temperature as follows [38, 39]:

$$\varepsilon \left(T\right)=s_{1}\left[1-\frac{1}{1+exp\left(s_{2}-s_{3}T\right)}\right]$$ (2)

$$\sigma \left(T\right)=r_{1}\left[1-\frac{1}{1+exp\left(r_{2}-r_{3}T\right)}\right]$$ (3)

The constants used in Eqs 2 and 3 introduced in Table 1. Due to the water evaporation during the MWA, dielectric properties of tissue decrease by rising temperatures [40]. It can thus be assumed that the change rate of dielectric properties with temperature is the same in both tumor and healthy tissues.

**Table 1:** The coefficients of the temperature dependence dielectric properties [38, 39, 41].

	Relative permittivity			Electrical conductivity		
	*s*_1_	*s*_2_	*s*_3_	*r*_1_	*r*_2_	*r*_3_
Healthy tissue	44.3	5.223	0.0524	1.80	6.583	0.0598
Tumor	54.8	5.223	0.0524	2	6.583	0.0598

### Biological heat transfer model

Pennes equation is widely adopted to model heat transfer in biological tissue by considering the interaction between vasculature and tissue [42]:

$$\rho C\frac{\partial T}{\partial t}=k\nabla^{2}T+\rho_{b}C_{b}\omega_{b}\left(T_{b}-T\right)+Q_{m}+Q_{ext}+Q_{E}$$ (4)

where *ρ*, *ρ*_*b*_, *C*, *C*_*b*_, k, *Q*_*m*_, and *ω*_*b*_ are the tissue density, the blood density (kg/m^3^), the specific heat capacity of tissue (J/kg∙K), the blood specific heat (J/kg.K), the tissue thermal conductivity coefficient (W/m∙K), the heat generated by metabolism (W/m^3^), which is negligible in comparison with the heat generated by MWA [32], and the blood perfusion (1/s). $Q_{ext}=\frac{\sigma {\left|E\right|}^{2}}{2}$ is the exterior heat source, which equals the heat of the resistance produced by the electromagnetic field. *Q*_*E*_, evaporation energy, is the energy needed to vaporize tissue water (see S1 Text). The liver tissue boundaries are assumed as the heat flux. In other words, the Neumann boundary condition can be employed in conjunction with the energy balance to give:

$$k\frac{\partial T}{\partial n}=h\left(T-T_{0}\right)$$ (5)

where h is the overall heat transfer coefficient for the region near the model boundary (h = 430W/m^3^∙K), and *T*_*0*_ is the surroundings bulk temperature [43].

Previous studies show that the tissue thermal conductivity varies with the temperature [35]. For more accuracy in the calculations, the following equation is used [10]:

$$k\left(T\right)=k_{0}+\Delta k\left[T-T_{0}\right]$$ (6)

where *k*_*0*_, Δ*k*, and *T*_*0*_ are respectively the baseline thermal conductivity, the change in *k* due to temperature, and the reference temperature at which *k*_*0*_ has been measured, which is in this case similar to the biological temperature.

Bio-heat transfer in living tissues depends on the blood perfusion via the vascular networks [44]. The difference in temperature between blood and tissue through which it flows causes convective heat transfer. Therefore, the blood perfusion rate is a function of temperature and is represented as follows:

$$\omega_{b}=0.000021\;T+0.0035$$ (7)

### Tumor shrinkage model

The effect of thermal ablation on tissue shrinkage has been recently considered in many studies [45–47]. Liu et al. [48] studied the effect of MWA on tissue contraction by numerical and experimental models. Their results show that over 50% of volumetric contraction occurred at the temperature over 102.1°C. Liu and Brace [49] analyzed six ex vivo bovine liver samples during MWA by using intraprocedural computed tomography (CT) imaging. Their results indicate that the ablation zone in post-treatment is significantly decreased by approximately 45%.

A linear elastic model and thermal strain can describe volume deformation due to the thermal ablation:

$$E_{el}=E_{tot}-E_{el}$$ (8)

The thermal strain was defined as:

$$E_{th}=\alpha_{t}\left(T-T_{ref}\right)$$ (9)

where *T*_*ref*_ is the initial temperature (K) and *α*_*t*_(*K*^−1^) is the thermal expansion coefficient that calculated as follows [48]:

$$\alpha_{t}=-\frac{0.03.TTI^{0.4684}}{T-285}$$ (10)

TTI is the temperature-time integration function, and it is evaluated by comparing with experimental data [48]:

$$TTI\left(T,t\right)=\{\begin{array}{l} \;0\;,\;\left(12{}^{\circ}C\leq T\right)\\ \;0.1573\;\int_{0}^{t}T-T_{0}dt,\;(12{}^{\circ}C<T\leq 44.1{}^{\circ}C)\\ \;0.3011\;\int_{0}^{t}T-T_{0}dt,\;(44{}^{\circ}C<T\leq 102.1{}^{\circ}C)\\ \;0.54163\;\int_{0}^{t}T-T_{0}dt,\;(T>102.1{}^{\circ}C) \end{array}$$ (11)

### Cell death model

In the present study, to investigate the effect of heat on the cell's condition, the cell death model developed by O’Neill et al. [33] is employed. This model illustrates the cell death process under temperature gradient by using coupled ordinary differential equations (ODEs), in which cells are divided into destroyed, denatured, or killed states. There is a vulnerable state located between the living and dead states, and it is a result of the direct observation of the cell’s response to thermal treatments. With consideration of the vulnerable state, the results of the model are more consistent with experimental data [32]. The transition from living to vulnerable describes the initial damage leading to an injured state and is represented by a forward rate constant, *k*_*f*_, while a backward rate constant, *k*_*b*_, describes a self-healing process from vulnerable to a fully functional alive state. Once past a critical point, the cell progresses to a dead (*D*) state, after which the process is irreversible.

$$A\overset{k_{f}}{\underset{k_{b}}{⇄}}V\overset{k_{f}}{\to }D$$ (12)

*A*, *V*, and *D* are respectively fractions of alive, vulnerable, and dead cells which are defined as the following system of equations [33]:

$$\frac{dA}{dt}=-k_{f}A+k_{b}\left[1-A-D\right]$$ (13)

$$\frac{dD}{dt}=k_{f}[1-A-D]$$ (14)

$$k_{f}=\bar{k_{f}}e^{\frac{T}{T_{k}}}[1-A]$$ (15)

in which $\bar{k_{f}}$ is a scaling factor (1/s) and *T*_*k*_ is a parameter setting the rate of the exponential increase with temperature.

The presented model allows the data fitting over a wider range of thermo-therapeutic temperatures, including the early stage, when cells exposed to the heat for the first time. The cell viability, (V+D), is utilized to determine the lesion size. This study is the first application of the presented model to analyze MWA for extracting the size and shape of the lesion, monitoring the effect of tumor shape and size, and investigating the side effects.

### Model geometry

**Fig 1:**
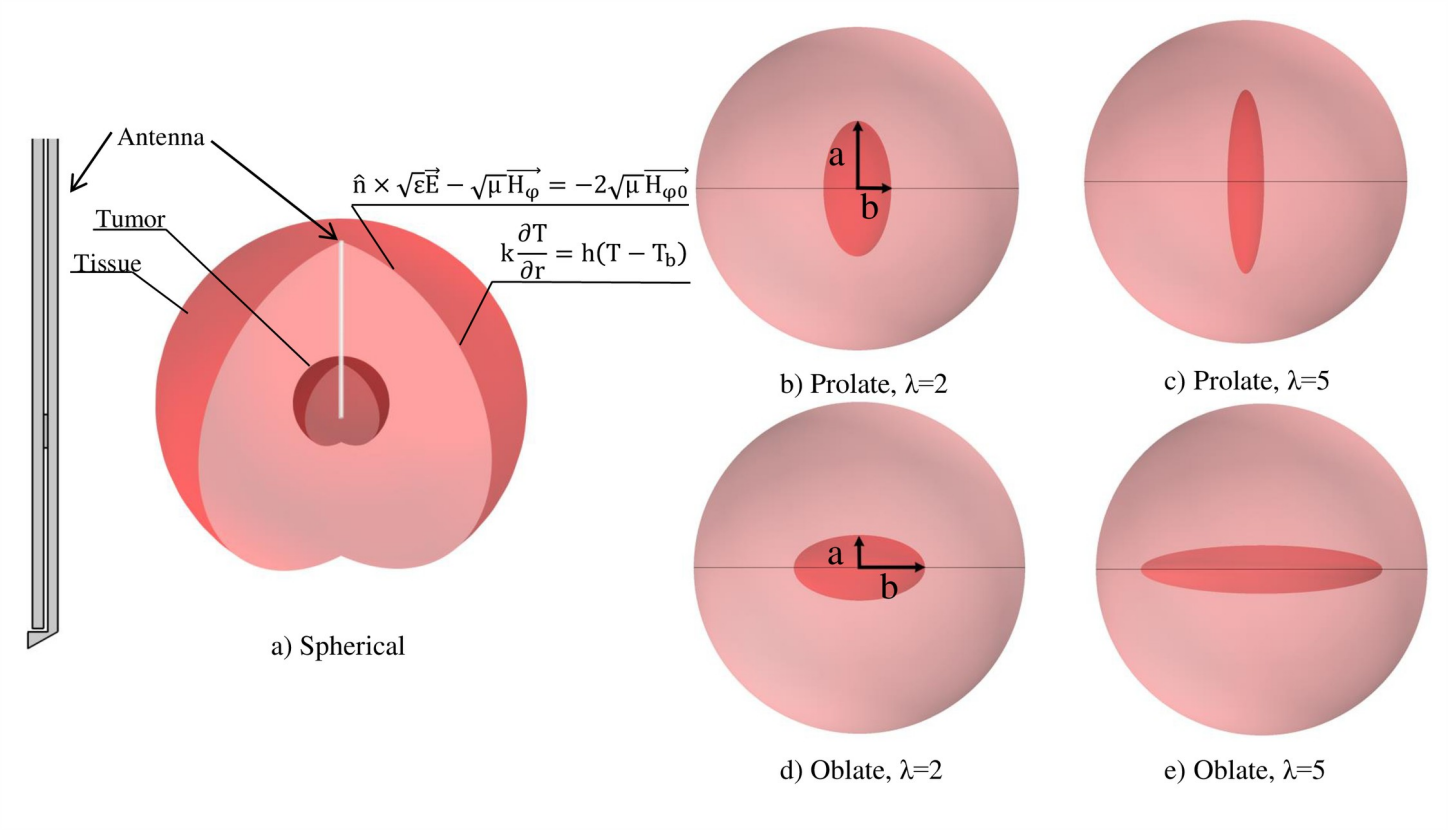
Boundary conditions and the geometry of the present study with different shapes of the tumor. Since all of the tumors are not necessarily spherical, different shapes of the solid tumors are considered in this study. In all oblate, prolate, and spherical shapes, the volume of the tumors are constant. MWA coaxial antenna is placed at the center of the tumor. Thermal and electrical boundary conditions are applied to the wall of the tissue.

The tumor nodules have different shapes and sizes in various cancer types, such as breast, prostate, and liver to name a few. According to the literature [50–54], three shapes of the tumor (i.e., spherical, oblate, and prolate) are considered to cover most of the shapes. The geometry of the present study with different shapes of the tumor is shown in Fig 1. The dimensions of the antenna are similar to Yang et al. [55]. The initial geometry consists of the spherical tumor with a particular radius *r*, the second one is prolate, and the last one is the oblate ellipsoid shape. The ratio of the minor-to-major axes of both prolate and oblate ellipsoids is demonstrated by *λ* (or *a*/b). All of the tumor shapes shown in Fig 1 have a constant and equal volume compared to the spherical tumor volume with radius *r*.

### Numerical approach

All geometries were created and meshed in COMSOL Multiphysics. The coupled nonlinear set of governing equations mentioned in previous sections and also the boundary conditions were implemented using the FEM. A segregated approach was employed for solving the equations. Due to the length of MWA treatment (typically ranging from 10 to 20 min), a transient model was applied with the 0.1 s time-step and 0.001 relative tolerance for solving bio-heat transfer, cell death model, and tissue shrinkage equations. A frequency study used to solve electromagnetic wave equations. A drop of four orders of magnitude in the residuals was selected as a convergence criterion. For the grid independency, 81,253 tetrahedral elements with 1,3581 triangular with average element quality of 0.7643 were chosen (the grid independency presented in S1 Text in detail). A 7700 HQ intel 7^th^ generation CPU @ 2.8 GHz with an 8 GB memory RAM was used for the simulations.

The parameters of biological materials used in numerical simulations have been summarized in Table 2 [10, 35]. Also, these parameters are being assumed constant in the simulation.

**Table 2:** Parameters of the biological materials [10, 35].

Parameter	value
Properties of tissue	
Density, *ρ*_*tissue*_	1079 kg/m^3^
Thermal conductivity, *k*_*0tissue*_	0.52 W/m^º^C
Specific heat, *C*_*tissue*_	3540 J/kg^º^C
Properties of tumor	
Density, *ρ*_*tumor*_	1040 kg/m^3^
Thermal conductivity, *k*_*0tumor*_	0.57 W/m^º^C
Specific heat, *C*_*tumor*_	3960 J/kg^º^C
Properties of the blood	
Density, *ρ*_*b*_	1060 kg/m^3^
Thermal conductivity, *k*_*b*_	0.5 W/m^º^C
Specific heat, *C*_*b*_	3600 J/kg^º^C
Blood temperature, *T*_*b*_	37^º^C
Rate of change of thermal conductivity, Δ*k*	0.001161 W/mK

### Validation

**Fig 2:**
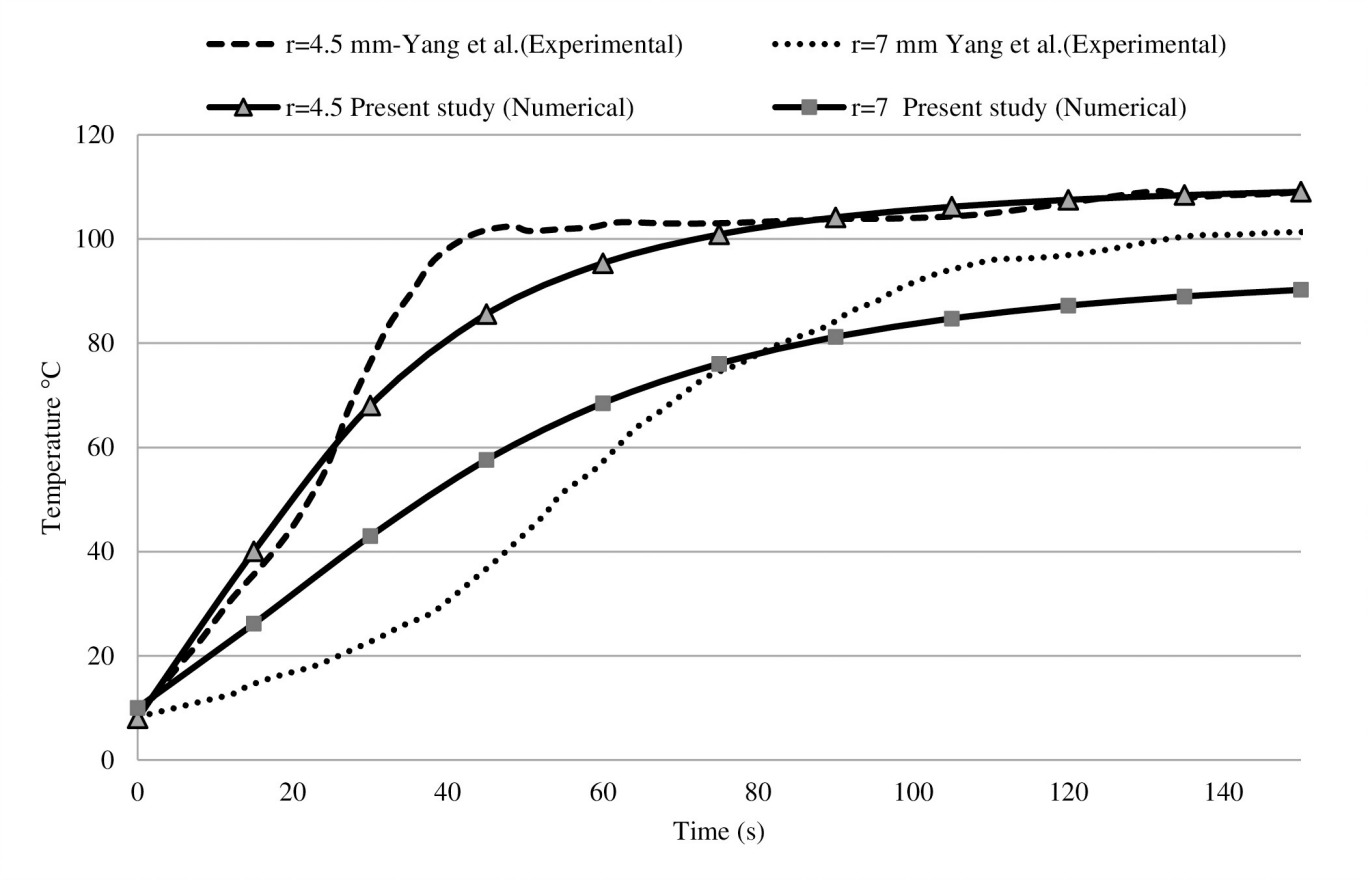
The validation of the current study’s results with the experimental work of Yang et al. [55]. As same as an experimental test, MWA simulated with 75 W input power and frequency of 2.45 GHz. Temperature increment during 150 s MWA is monitored at two point. Our simulation has 3.2% and 6.3% difference with experimental data at 4.5 mm and 7 mm away from the antenna, respectively.

To check out the accuracy of the parameters and equations of the current study, the simulation results are compared with the previously published studies of Yang et al. [55] and Sun et al. [56]. The microwave signal with 75W and 2.45 GHz applied to the liver tissue of 5°C as its initial temperature. For monitoring temperature during the treatment, two positions of 4.5 mm, 7 mm were considered radially away from the antenna slot. Results of the present study, as illustrated in Fig 2, demonstrated a good agreement with literature; accordingly, they have a 4.75% difference compared to the experimental and numerical results reported by Yang et al. [55], on average.

**Fig 3:**
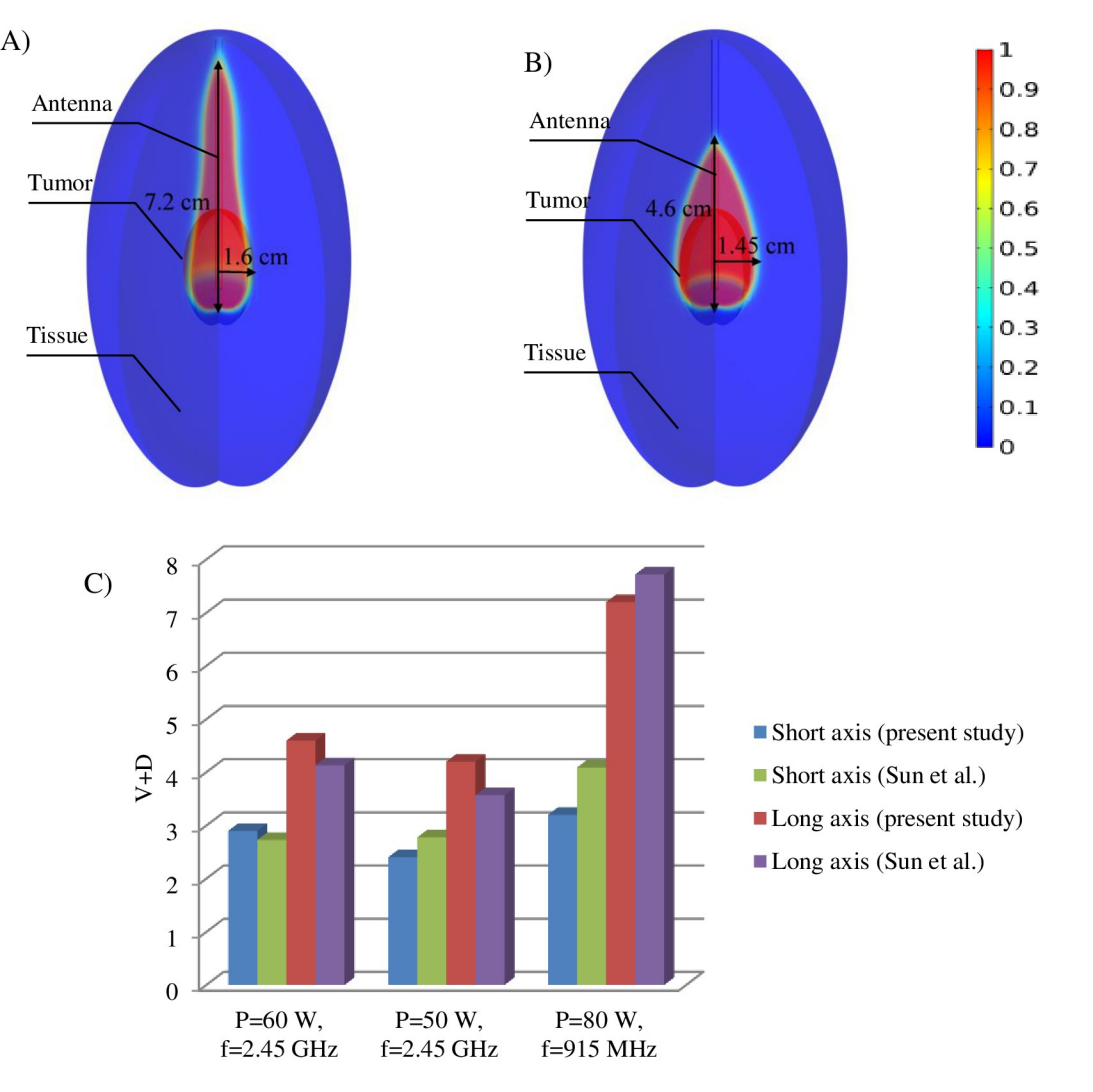
Validation of numerical results with a previously published study. (A and B) Ablation zone (V+D) calculated by numerical simulation by using the three-state model. (P = 80 W, f = 915 MHz). In the 50 W input power, by increasing frequency to 2.45 GHz, the long and short axis of the ablation zone decreases to 4.65 cm and 1.45 cm, respectively (B). The dimension of the ablation zone has a good agreement with experimental data. (C) Dimensions of the ablation zone calculated by the present numerical simulation and experimental results achieved by Sun et al. [56].

**Fig 4:**
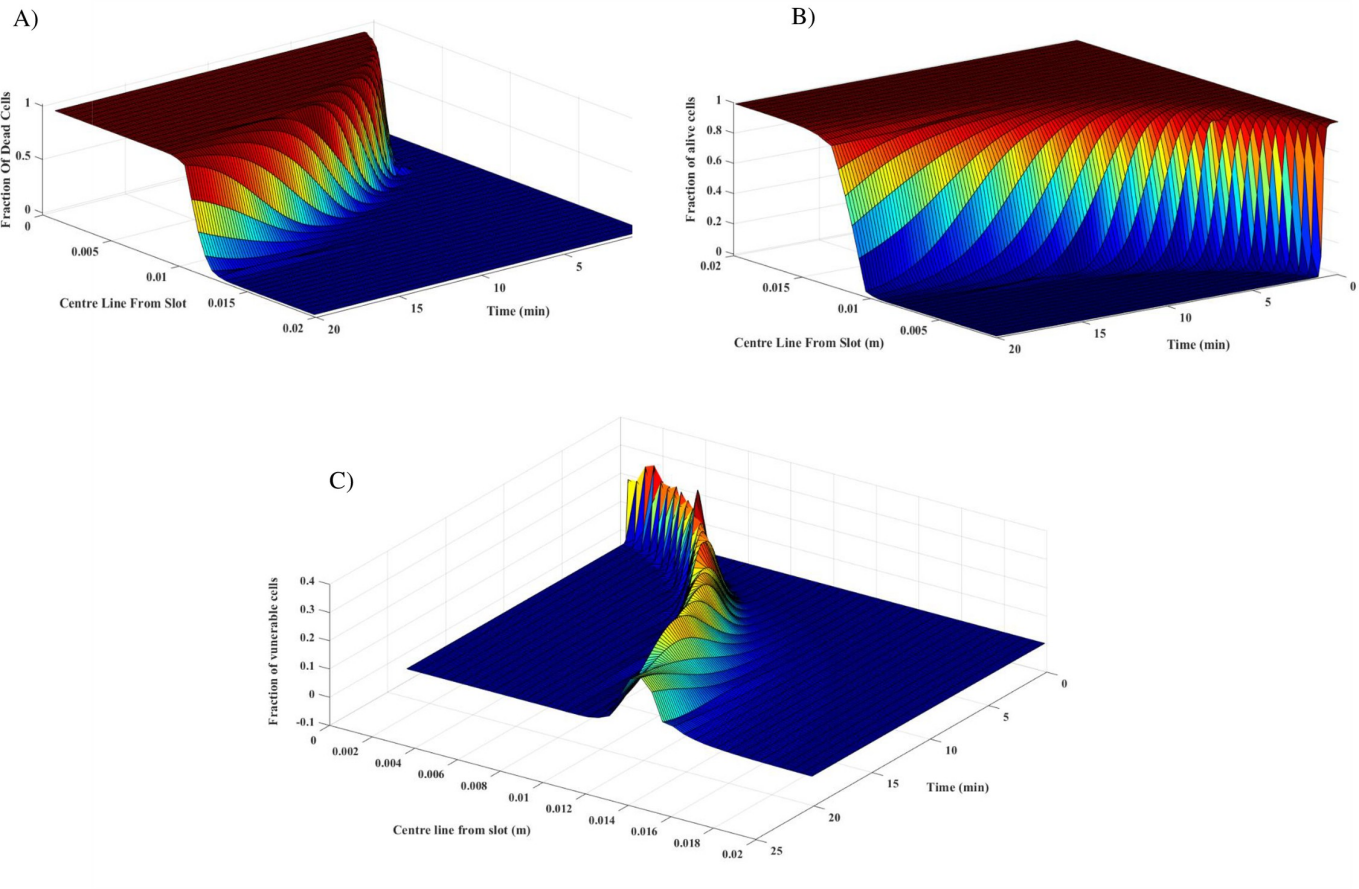
The variation of percentage of cells in tumor radius length. The three-state mathematical model describes an accurate cell’s response to the temperature during treatment (P = 45 W and f = 2.45 GHz). The fraction of dead cells increases in the tumor by increasing temperature (A) and as expected, fraction of alive cells decreases in the ablation zone (B). Vulnerable is a transient state between alive and dead cells, and some of the cells remain in this state in any treatment time (C).

Fig 3 shows the lesion area at a different frequency and power values obtained by using the presented three-state mathematical model. To ensure the accuracy of the three-state model, the results are compared with the values measured by Sun et al. [56]. To calculate the region affected by the temperature gradient, the sum of the percentage of damaged and dead cells (D+V) were taken into account. In this study, the ablation zone dimensions obtained by numerical simulation are very consistent with the practical measurement. Fig 4(C) compares the short axis and the long axis of the ablation zone in numerical results of the present study and the experiment data extracted by Sun et al. [56]. Results demonstrated 12% and 9% difference in comparison with the literature [56] for the short axis and the long axis, respectively.

## Results

### Cell death process during thermal ablation

Fig 4 shows the percentage of alive, vulnerable, and dead cells at different distances from the slot over time in tumor and normal tissue for a tumor with a radius of 5 mm as a sample. The ablation zone expands during the treatment, as shown in Fig 4(A) and 4(B). After 5 minutes, all of the cancerous cells die, and the lesion meets the tumor borders. Fig 4(C) demonstrates the variation of the vulnerable cells during MWA. As the temperature increases, the percentage of vulnerable cells increases up to 60% of the total cells. By continuing treatment, these cells will also be destroyed.

### The effect of input power on treatment efficiency

**Fig 5:**
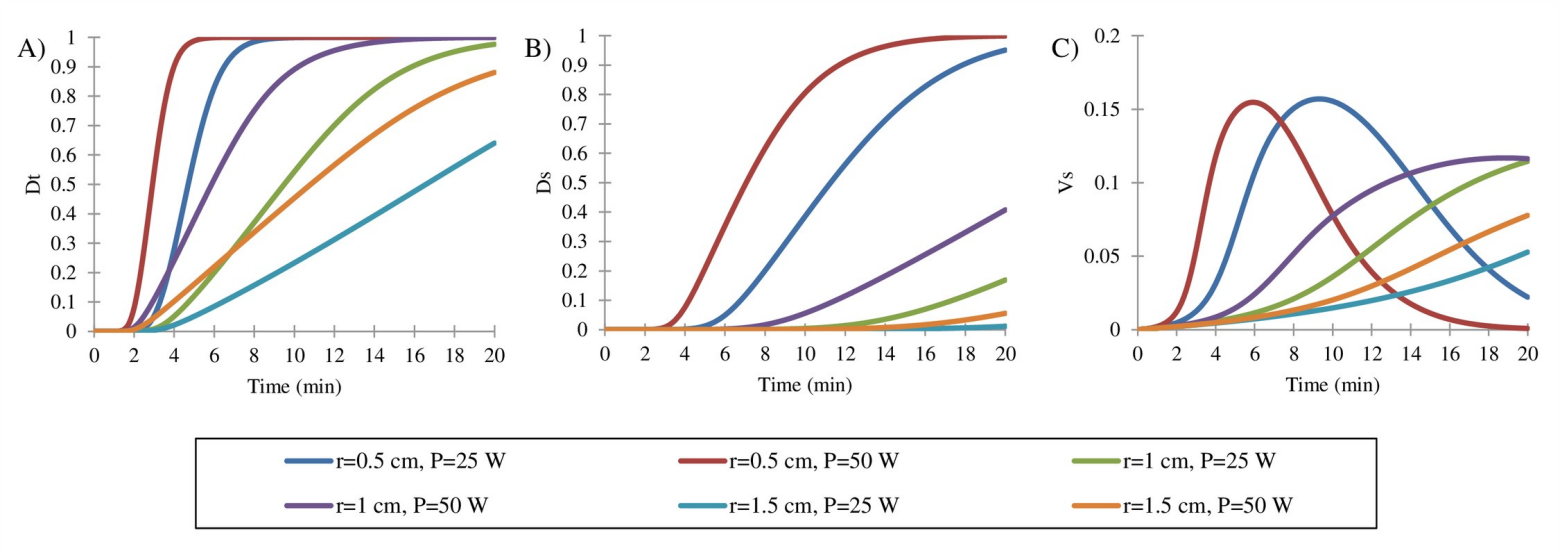
Effect of change in input power at different tumor sizes. (A) Mean fraction of dead cells in three tumor sizes. In smaller tumor (r = 0.5 cm and r = 1 cm), all of the cancer cells are ablated as treatment continues, but in the tumor with 1.5 cm, 21% of cancer cells remain alive, even by increasing the input power to 50 W. Treatment efficacies are related to the collateral damage directly, therefore mean fraction of dead cells in normal tissue around the tumor is presented to restrict treatment duration by allowable side effects (B). Due to this issue, the ablation of all parts of the tumor is not possible in all cases. At any treatment time, a fraction of cells remain in a vulnerable state. These cells do not necessarily die after treatment; therefore, the three-state mathematical model helps to estimate treatment efficacy more accurately.

Fig 5 indicates the effect of the input power in various tumor sizes. The allowable fraction of cell death in the normal tissue is assumed to be 10%, on average. The cell dead processes occur faster in smaller tumors and higher input power. The mean percentage of cells killed in a 0.5 cm tumor radius reached their maximum value at both 25 W and 50 W input power. In the case of a 1.5 cm radius tumor, Fig 5(A) indicates that at 50 W, 85% of cancer cells killed, which is 21% larger than 25 W input power as its mean fraction of dead cells is 64%. At the allowable side effect, the mean percentage of vulnerable cells did not change in different cases.

### The effect of tumor shape on thermal ablation

**Fig 6:**
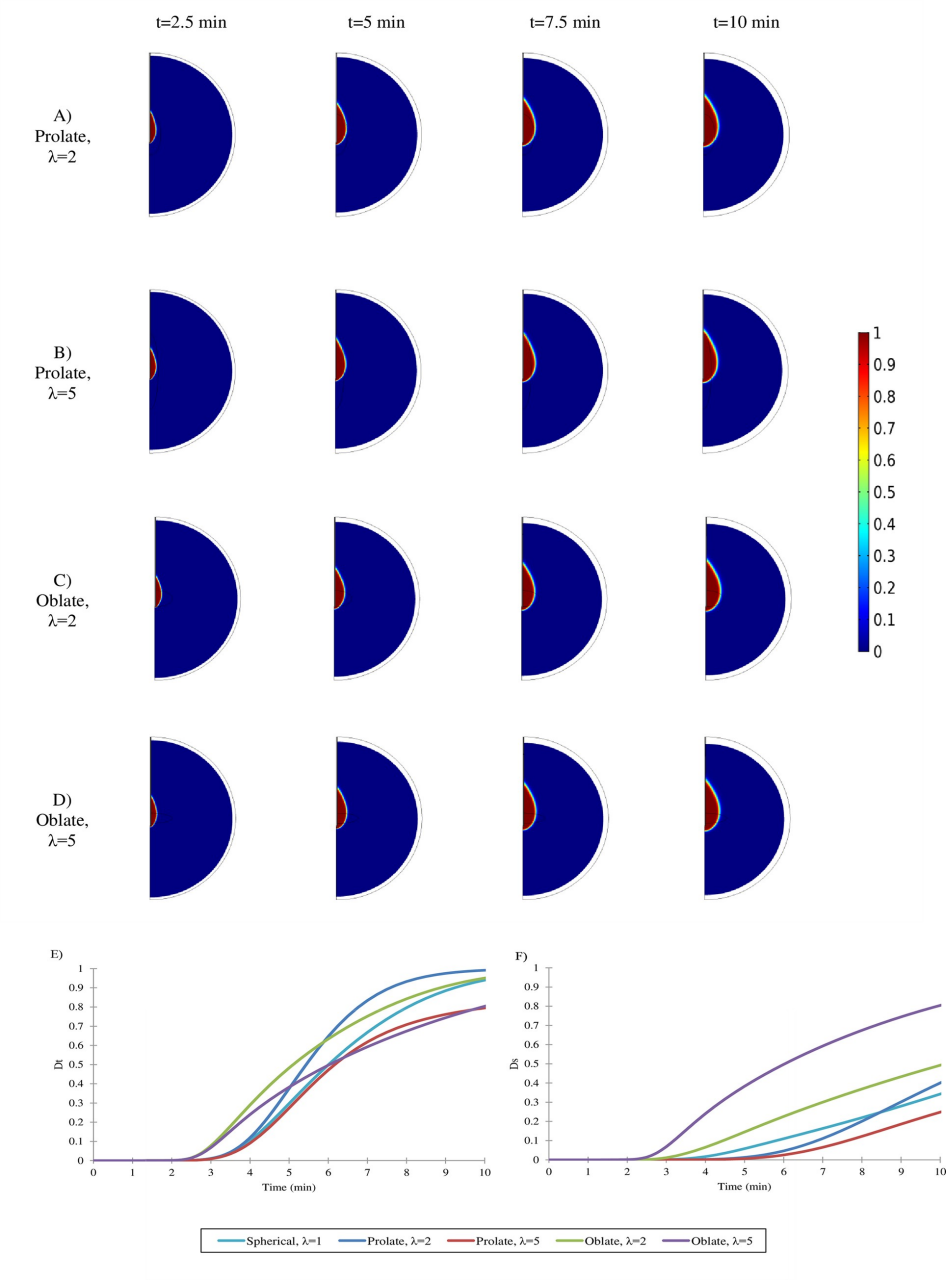
The profile of the ablation zone according to the fraction of dead cells. The expansion of the ablation zone during the MWA process are shown in different tumor shapes and sizes (A-D) (P = 20 W and f = 2.45 GHz). (E) The average percentage of dead cells in λ = 5 oblate and prolate tumor, all of the tumor will be ablated, but 50% and 58% of normal cells die in prolate and oblate shape, respectively (E and F). Treatment of oblate tumors is more difficult. The ablation and due to the side effects, the whole tumor ablation is not possible. In the case of the prolate shape, if allowable mean percentage of dead cells in healthy tissue considered 10%, 77%, and 68% of the tumor will be ablated in λ = 2 and λ = 5. Therefore, in the same shape, MWA is more difficult by increasing length ratio.

To study the impact of tumor shape in treatment, two different types of oblate (flattened) and prolate (elongated) tumor shapes with two different aspect ratios (λ) are assumed. To compare accurately, tumor volume in all cases is considered equal to spherical tumors with radius r = 0.5 cm. Thermal ablation is applied for different tumor shapes in the same condition. Due to the tissue contraction, the tissue lost around 50% of its volume after treatment (Fig 6(A)–6(D)). The rate of cell death is demonstrated in Fig 6(E) and 6(F). In tumor with λ = 2, 40% and 77% of tumor ablated in oblate and prolate shape, respectively. In the case of λ = 5, 30% and 68% of tumor cells killed in oblate and prolate shape, respectively.

### The effect of frequency on the tumor shape

**Fig 7:**
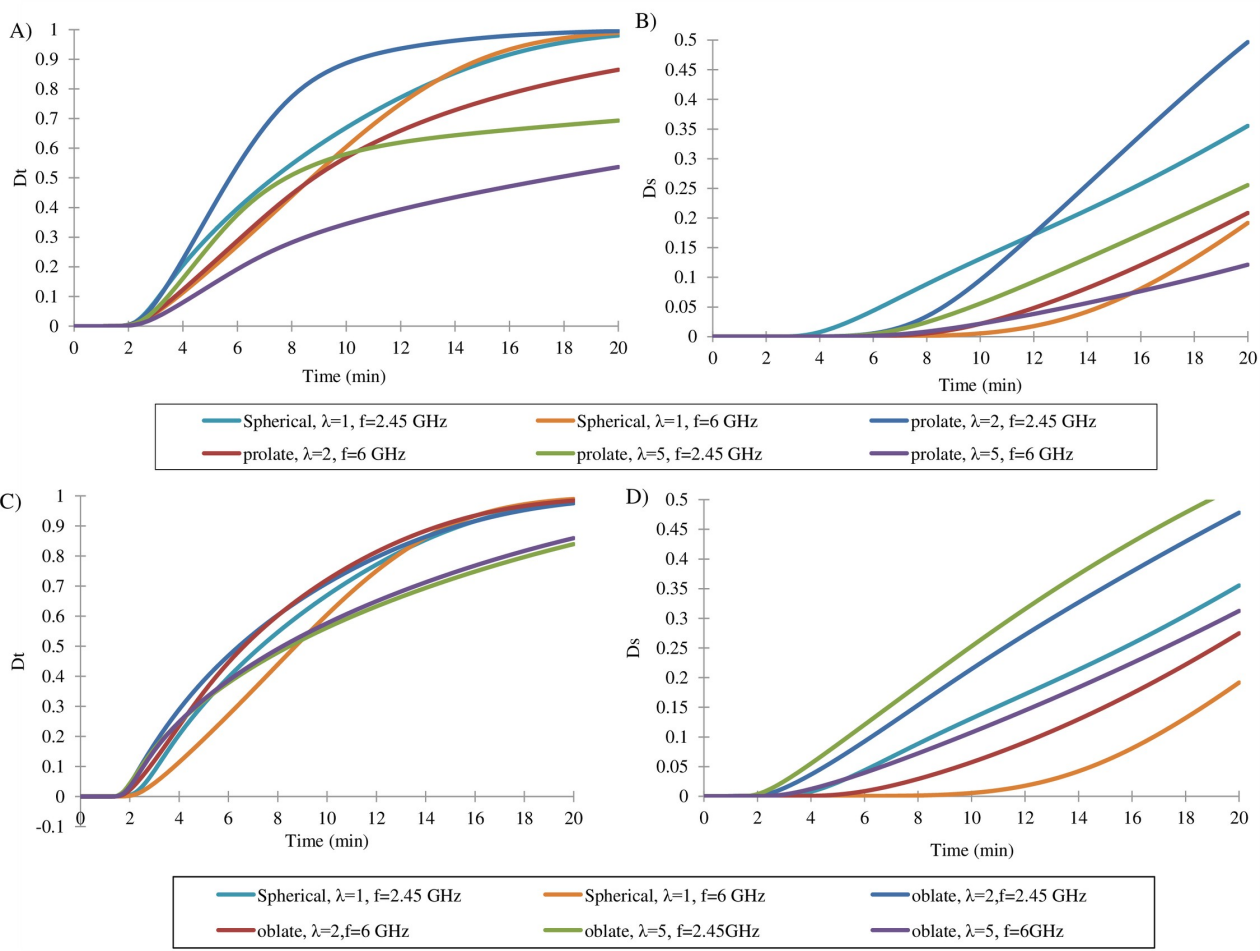
The average percentage of dead cells inside and around the tumor at frequencies of 2.45 GHz and 6 GHz. By increasing frequency from 2.45 GHz to 6 GHz, the mean fraction of dead cells in the prolate tumor decreased to 11% and 12% at λ = 2 and λ = 5, respectively (A). (B) Mean fraction of dead cells around the prolate tumor; that shows the side effects during MWA. Maximum allowable collateral damage considered 10%, on average. As well as result from Fig 7, the treatment of oblate tumors is more difficult, but by increasing frequency from 2.45 GHz to 6 GHz, the treatment outcome improved by 28% on average (C and D).

Fig 7 illustrates the effect of low and high frequency in MWA in different tumor shapes. Applying 2.45 and 6 GHz frequency results in 86% and 75% of tumor ablation in prolate shapes (λ = 2), and 62% and 50% in prolate shapes (λ = 5), respectively. In the oblate-shape tumor by applying MWA with higher frequency, for λ = 2 and λ = 5, the mean percentage of dead tumor cells has increased 35% and 21%, respectively (Fig 7(C)). In the case of spherical tumors, 60% and 90% of cancer cells killed with 2.45 GHz and 6 GHz MWA, respectively. In all comparisons, D_s_ = 10% is considered as an allowable cell death percentage in healthy tissue.

### The effect of using a double slot antenna on treatment efficiency

**Fig 8:**
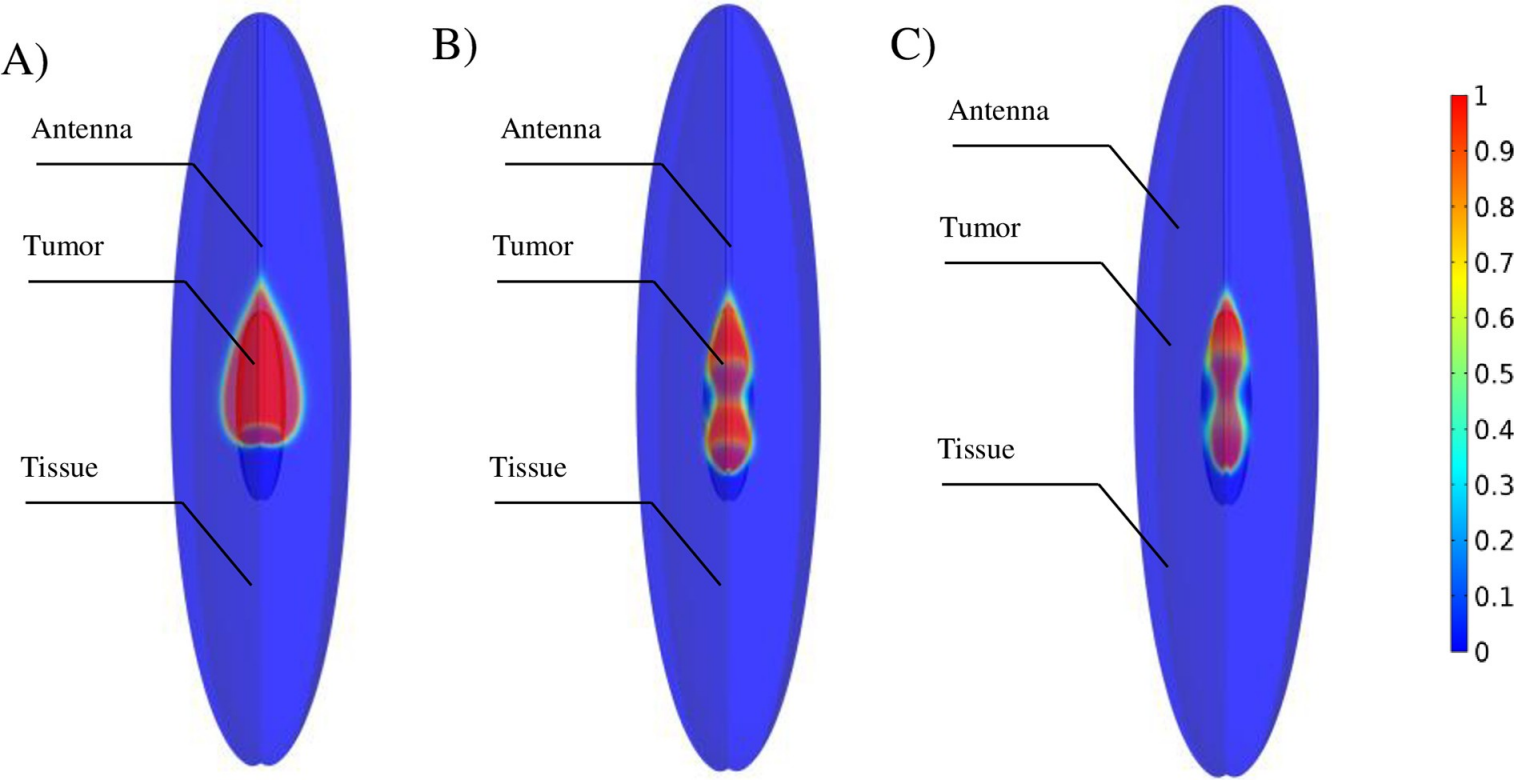
Tumor ablation with a single slot antenna and double slot antenna. MWA applied to the same tumor and the same condition by three different antennas (λ = 5, *f* = 2.45 GHz, P = 70W). By using a single slot antenna, around 76% of the tumor is ablated, but the ablation zone is expanded to the surrounding healthy tissue(A). In the case of using a double slot antenna, the ablation zone could be further adjusted into the tumor area by optimizing slot to slot distance (B and C).

In Fig 8, the effect of single and double slot antenna on ablation of prolate tumor is compared. In the case of using a single slot antenna, the slot should be placed in the center of the tumor. However, in the case of using a double slot antenna, the results showed that the bottom slot should be placed below the center of the tumor at the distance of *b*/2 [24]. In the case of using a single slot antenna, 76% of cancer cells are killed on average (Fig 8(A)). By using double slot antenna, 96% and 89% of the tumor is ablated when slot to slot distance is considered *L* = *b* and *L* = 1.4*b*, respectively.

## Discussion

The goal of the current study is to select the effective parameters such as frequency, power, and ablation time for different tumor geometries to obtain the treatment with minimum collateral damage. The differences in the shape and size of lesion and tumor have an important effect on treatment efficacy and side effects. Using a three-state mathematical model helps to accurately track the cells function under increasing temperature, especially by considering the vulnerable cells. To observe the damaged cells in healthy tissue, the percentage of dead cells is monitored in the area around the tumor. To have a correct comparison between various tumor shapes, the volume of this region is considered constant in all shapes. In the case of different tumor sizes, the volume of the region around the tumor could not be equal; therefore, a fixed point was considered 2.5 mm radially away from the tumor border to monitor the changing percentage of the destroyed cells.

The cell death rate due to temperature is shown in Fig 4. Extra treatment time causes more damage to the normal tissue; therefore, optimal treatment time must be applied for different tumor shapes and sizes. When the temperature reaches the critical value, previous studies considered alive cells as dead ones [10, 35]. However, by increasing temperature to the threshold temperature, the cell death process begins and more time needed to kill all cancer cells (see S2 Fig). In this study, the three-state mathematical model is implemented in order to estimate the size and the shape ablation zone. Vulnerable state, which is a state between alive and dead states, can consist of 30% of the total cells in their maximum value (Fig 4(C)); therefore, they can have a noticeable effect on collateral damage and treatment efficacy. It is worth mentioning that vulnerable cells may be destroyed or restored after treatment.

Previous studies have shown that the dimensions of the ablation zone have increased by power, but the effect of tumor size has not investigated [17]. An analysis was performed to show how tumor size could be a decisive factor in MWA. With reference to S3(A) Fig, the fraction of killed cancer cells decreased after thermal therapy in the same condition. To better analyze the relationship between the power, tumor size, and treatment outcome, a comparison is performed in the same side effects (*D*_*s*_ = 10%). In tumors with a radius of less than 1 cm, applying more power causes tumor ablation in less time, and the percentage of damaged cells decreases slightly (about 3%). In the treatment of larger tumors (*r* = 1.5 cm), by using low power, only 60% of the tumor can be eliminated. Using high power ablation obtains better results, and a larger area of the tumor can be destroyed. On the other hand, side effects do not change significantly because, in larger tumors, the healthy tissue is farther away from the antenna. Therefore, increasing the power for tumors with a radius of less than 1 cm is not a good choice. In this case, any errors in choosing the correct treatment time cause more collateral damage compared to the lower power, and also at the optimum time, side effects are approximately equal in both cases. In large tumors (r≥1.5 cm), high power can destroy a higher percentage of the cancer cells at an allowable side effect range. Therefore, the higher input power was not a necessarily better option in all cases, and this parameter must be optimized according to the tumor size in clinical treatments.

Because all of the solid tumors are not spherical, the effect of tumor shape is studied in Fig 6. To assess the side effects, the volume of the surrounding tissue in all cases is considered eight times the tumor volume. The level of consistency between tumor shape and dimensions of the lesion has a significant effect on treatment efficiency and collateral damage. The lowest consistency belongs to oblate tumors because the ablation zone growth along the antenna is in the shape of teardrop (especially in the case of using lower frequency) (Fig 6(A) and 6(B)). Fig 6(F) shows the mean percentage of dead cells around the tumor to monitor side effects. If the allowable fraction of cell death in the area around the tumor is assumed to be 10% on the average, for λ = 2 and λ = 5, 40% and 30% of cancer cells have died in oblate shape (Fig 6(E)), respectively. In the case of prolate shapes, these fractions change to 77% and 68% for λ = 2 and λ = 5, respectively. Therefore, with an increase in λ, the side effects are increased, and smaller percentages of the tumor can be treated. Generally, in the same λ, treating more massive tumor are more complicated (for more information, see S4 Fig).

The lesion profile is a function of frequency, and this fact can contribute to better treatment. As well as treating tumors with higher λ is more complicated. In previous studies, the effect of frequency was examined without any attention to the tumor shape [14, 15]. By using a lower frequency in prolate shape, better results obtained so that the average percentage of the dead cells is increased (Fig 7(A)). In constant side effects, applying lower frequency causes a 11% and 12% increase in the average cell death fraction in the tumor for λ = 2 and λ = 5, respectively. In spherical tumors, using a high frequency provides better results, and in constant side effects, up to 90% of cancer cells are lost. However, previous studies mentioned high-frequency MWA as a beneficial method [10, 15]; the results of our study illuminate that it is not necessarily true in all cases. Best MWA frequency should be selected by noticing the tumor shape to achieve the best treatment efficacy. As a consequence, frequency and power, which are the most important parameters in MWA, should be considered according to the tumor shape and size in order to achieve the best treatment with minimum collateral damage.

The ablation of the oblate shape tumor is more complicated than that of the spherical shape, especially at a higher λ (Fig 7(C)). Given the fact that the treatment of oblate tumors with high λ is more difficult, the use of high frequencies is an effective solution. Therefore, increasing the frequency raises the number of the average dead cells inside the tumor and reduces side effects at the oblate shape. However, in prolate shape, using a lower frequency leads to improvement in treatment by 12%. This implies that an increase in frequency does not essentially improve the treatment; therefore, the tumor shape has a significant impact on better treatment.

In the literature [21, 22, 35], the double slot antenna has been used to increase and concentrate on the temperature. The distance between double slots was optimized based on the more effective treatment of spherical tumors. In the present study, the double slot antenna is suggested for the treatment of prolate tumors because treat non-spherical tumors are more challenging. The distance between two slots (*L*) is the most critical and challenging parameter that affects the shape of the ablation zone, and it should be optimized according to the tumor geometry. As *L* increases, the ablation zone expands within the tumor (Fig 8(C)), and a dramatic increase in this parameter experiences a rise in the side effects. By using an optimized double slot antenna, the ablation zone can be focused inside the borders of the tumor, and a larger volume of the tumor is destroyed (S5 Fig). Due to changes in the dimensions of the ablation zone, the use of optimal antenna not only has a positive effect on the treatment of the spherical tumor, but also increases the side effects.

## Conclusion

In this research, a coupled three-state mathematical model, bio-heat, and electromagnetic equations were solved to investigate the effects of different sizes and shapes of the tumor in MWA. This is the first time that the three-state mathematical model has been used in MWA, and the effect of the treatment on various tumor shapes and sizes was considered. The volume of the tumor, along with the surrounding tissue that was lost during the treatment, was calculated. Various parameters were studied to eliminate the tumor with the fewest side effects. Results demonstrated that by increasing the size of the tumor, a lower percentage of cancer cells were killed and more side effects remained. The shape of the tumor also affects the treatment. Eliminating elliptic tumors is more difficult than eliminating spherical tumors by using MWA. Moreover, side effects in prolate shape are fewer than those in oblate shape. For tumors larger than 2 cm, increasing power has positive effects. The best frequency for treatment should be chosen according to the shape of the tumor. Otherwise, it just destroys the healthy tissue. For tumor with prolate and oblate shape, the upper and lower frequency is more suitable, respectively. This can increase treatment efficacy by 34%. For a better treatment of prolate tumors, a double slot antenna was used. The distance between the two slots was set to create a more spherical area. Results showed that the optimum distance between the two slots depends on the shape of the tumor, and *L*/*b* = 1 is optimum for prolate shape tumors. The results of the present study can be used to select the best method for achieving the best outcome of the treatment according to the shape and size of the tumors.

## Supporting information

S1 Fig Grid independency of the current study. Mesh independency was jugged by the temperature at 2.5 mm away from the slot during MWA. Mesh resolution was considered as acceptable when no significant difference between successive meshes was noticed in temperature at the selected point. This situation achieved in case 4.
(TIF)

S2 Fig The change of cells percentage in tumor radius length for spherical shape and size of 10 mm (*f* = 2.45 GHz, P = 40W). Three state mathematical cell dead models can describe cells' response to the temperature accurately. A transitive state that considered between alive and dead states helps to simulate the cell death process during MWA.
(TIF)

S3 Fig The effect of tumor size on MWA (*f* = 6 GHz, P = 50 W). The average percentage of dead cells are monitored during thermal ablation in three different tumor size (A). As well as, treatment efficiency depends on side effects directly, the fraction of dead cells is presented at a distance of 2.5 mm from the tumor wall (B). In the smallest tumor elimination of all the tumor is possible without excessive side effects, but in the 1 cm and 1.5 cm tumor side effects are increased to 15% and 35%, respectively. The fraction of vulnerable cells at a distance of 2.5 mm from the tumor wall is rising by increasing tumor size (C). By increasing tumor size, thermal ablation will be more difficult.
(TIF)

S4 Fig The treatment of oblate and prolate tumors by increasing tumor size (f = 6 GHz and P = 60 W). The mean fraction of dead cells presented for different tumor sizes on the same side effects (A). As the size of the tumor increases, the maximum fraction of dead cells are increased at the constant allowable collateral damage. Ablation time is an essential parameter for MWA and increases by increasing tumor volume (B).
(TIF)

S5 FigSlot to slot distance in double slot antenna according to the different tumor shape and size.The vertical axis shows the mean fraction of dead cells in the tumor and the longitudinal axis shows the ratio of the distance between slots to the tumor radius. Allowable side effects are considered 5% in the healthy tissue. In all cases, the best treatment result achieved at $\frac{L}{b}=1$.(TIF)

S1 Text (DOCX)
