# Supplementary figures and images for: Use of microwave ablation for thermal treatment of solid tumors with different shapes and sizes—A computational approach
Source: PLoS One. 2020 Jun 15;15(6):e0233219. doi: 10.1371/journal.pone.0233219 (PMC7295236; doi:10.1371/journal.pone.0233219)

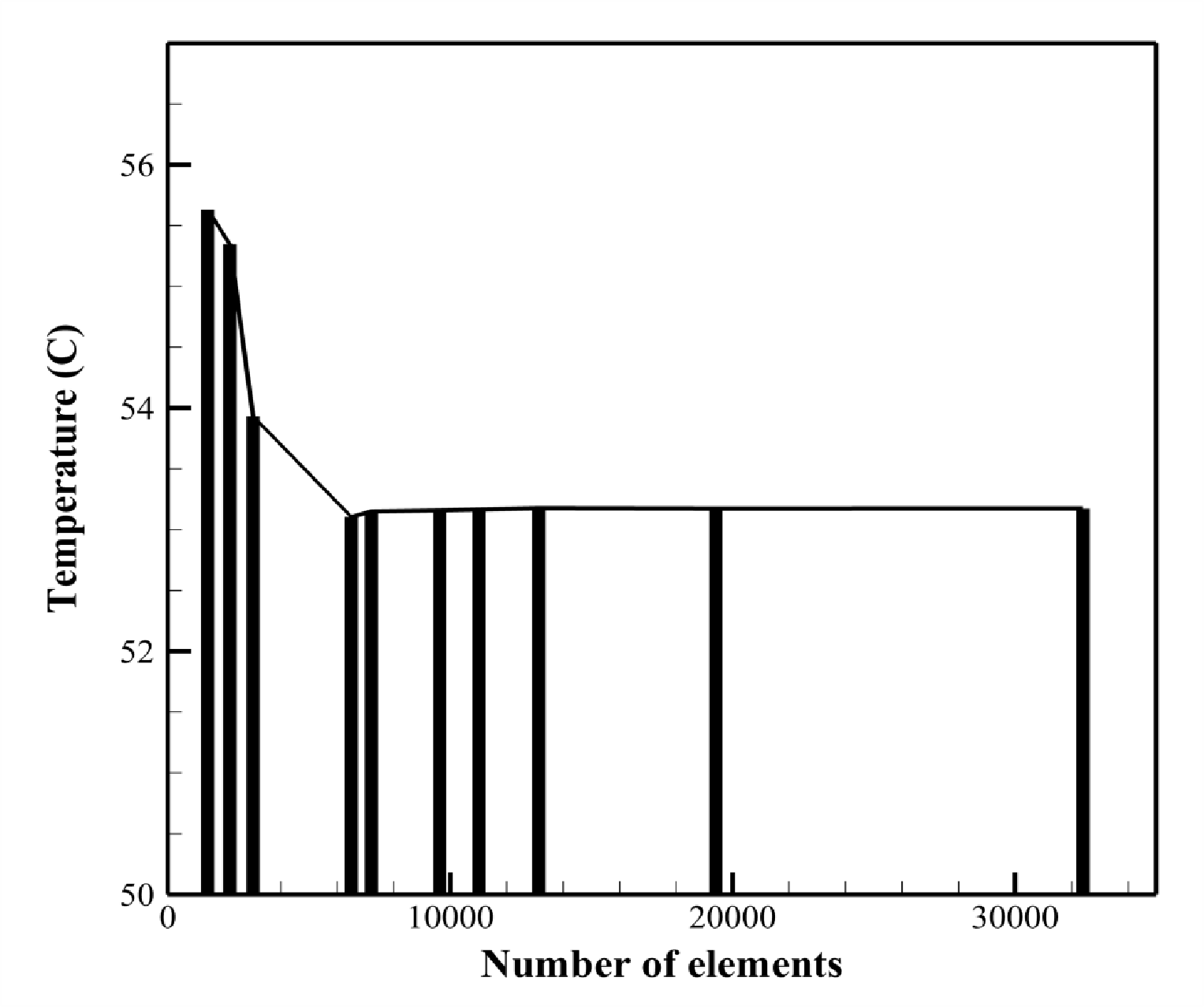

Supplement: S1 Fig — Mesh independency was jugged by the temperature at 2.5 mm away from the slot during MWA. Mesh resolution was considered as acceptable when no significant difference between successive meshes was noticed in temperature at the selected point. This situation achieved in case 4. (TIF) [file pone.0233219.s001.tif]

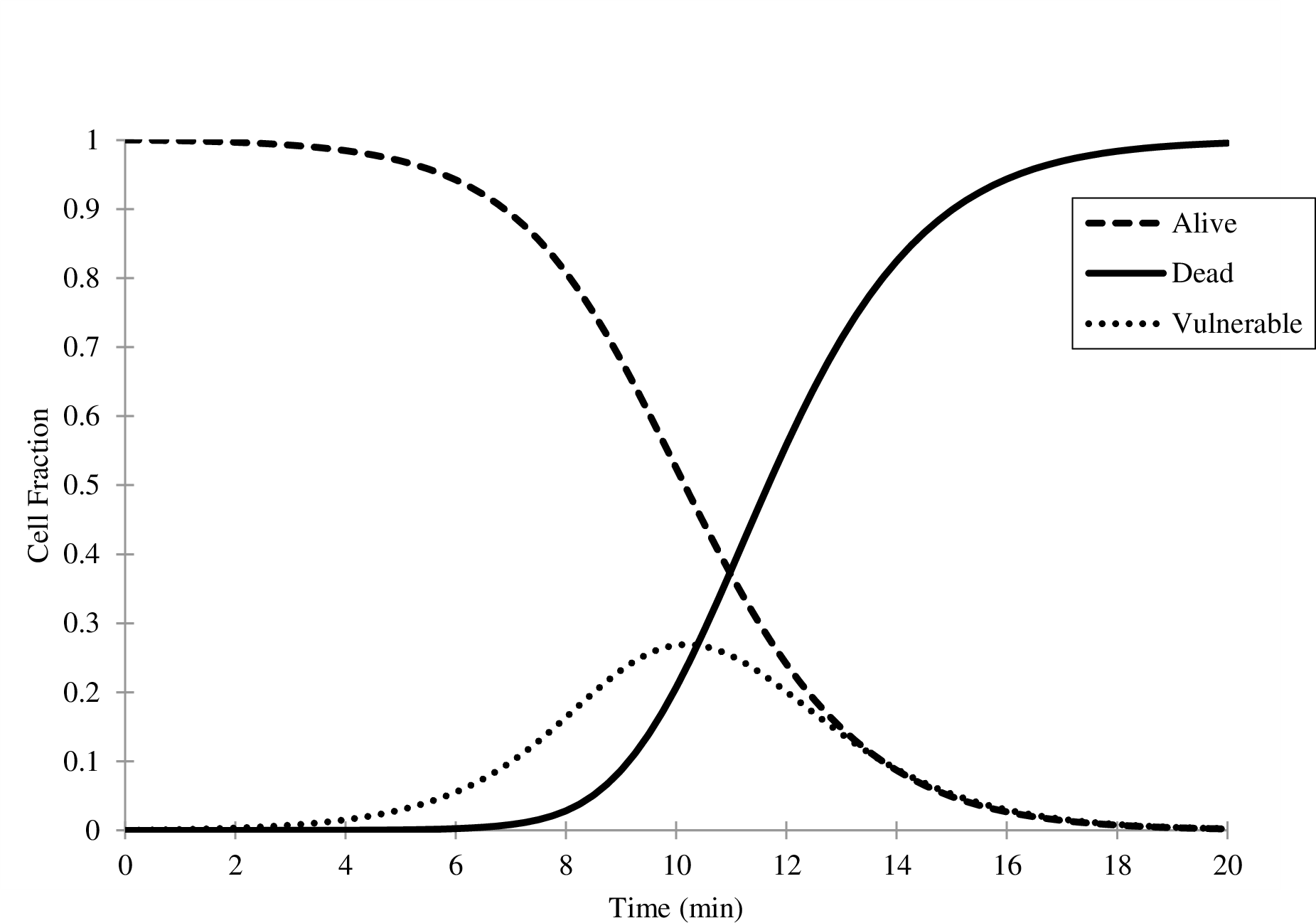

Supplement: S2 Fig — Three state mathematical cell dead models can describe cells' response to the temperature accurately. A transitive state that considered between alive and dead states helps to simulate the cell death process during MWA. (TIF) [file pone.0233219.s002.tif]

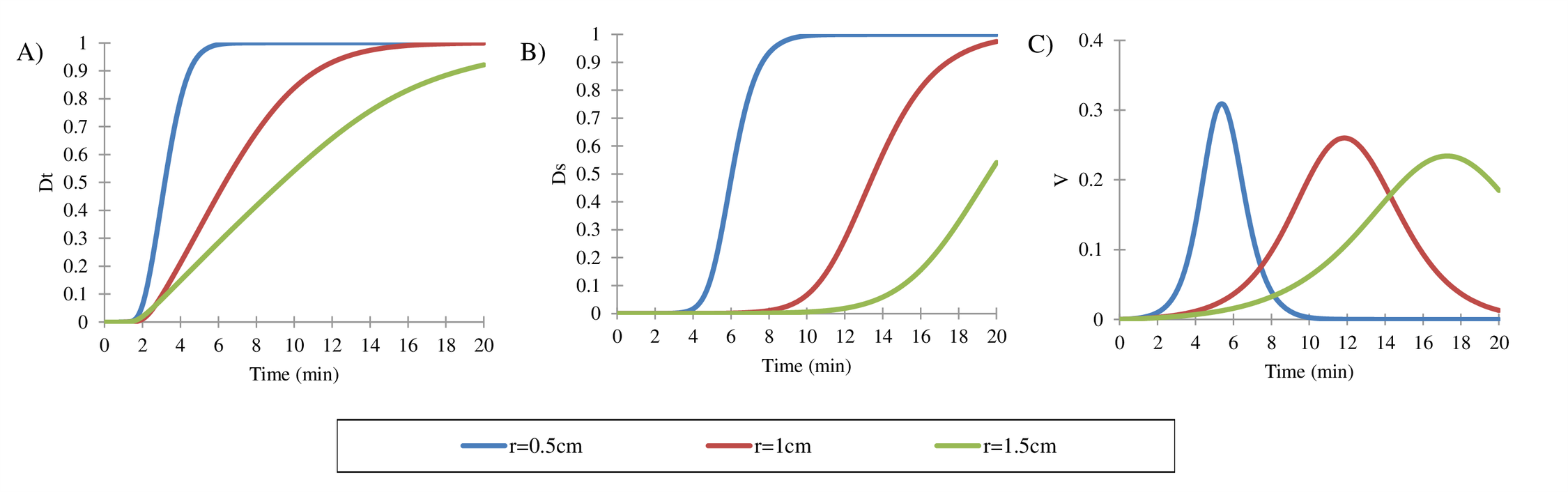

Supplement: S3 Fig — The average percentage of dead cells are monitored during thermal ablation in three different tumor size (A). As well as, treatment efficiency depends on side effects directly, the fraction of dead cells is presented at a distance of 2.5 mm from the tumor wall (B). In the smallest tumor elimination of all the tumor is possible without excessive side effects, but in the 1 cm and 1.5 cm tumor side effects are increased to 15% and 35%, respectively. The fraction of vulnerable cells at a distance of 2.5 mm from the tumor wall is rising by increasing tumor size (C). By increasing tumor size, thermal ablation will be more difficult. (TIF) [file pone.0233219.s003.tif]

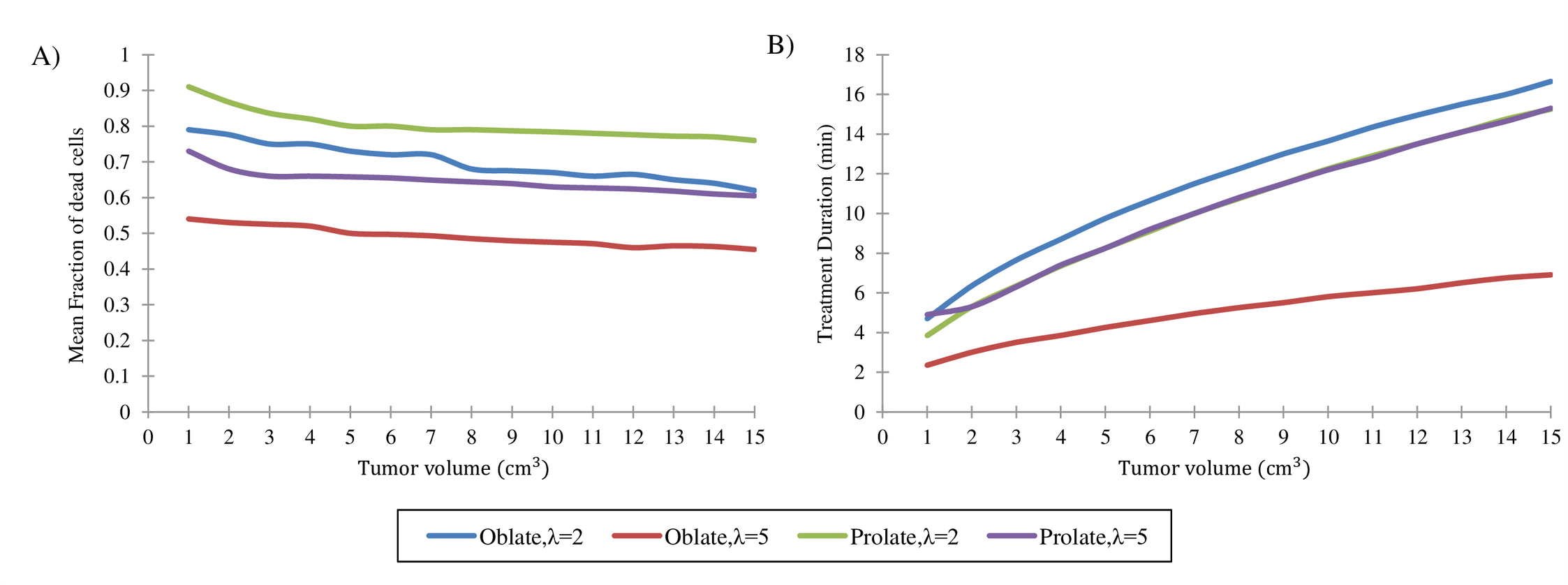

Supplement: S4 Fig — The mean fraction of dead cells presented for different tumor sizes on the same side effects (A). As the size of the tumor increases, the maximum fraction of dead cells are increased at the constant allowable collateral damage. Ablation time is an essential parameter for MWA and increases by increasing tumor volume (B). (TIF) [file pone.0233219.s004.tif]

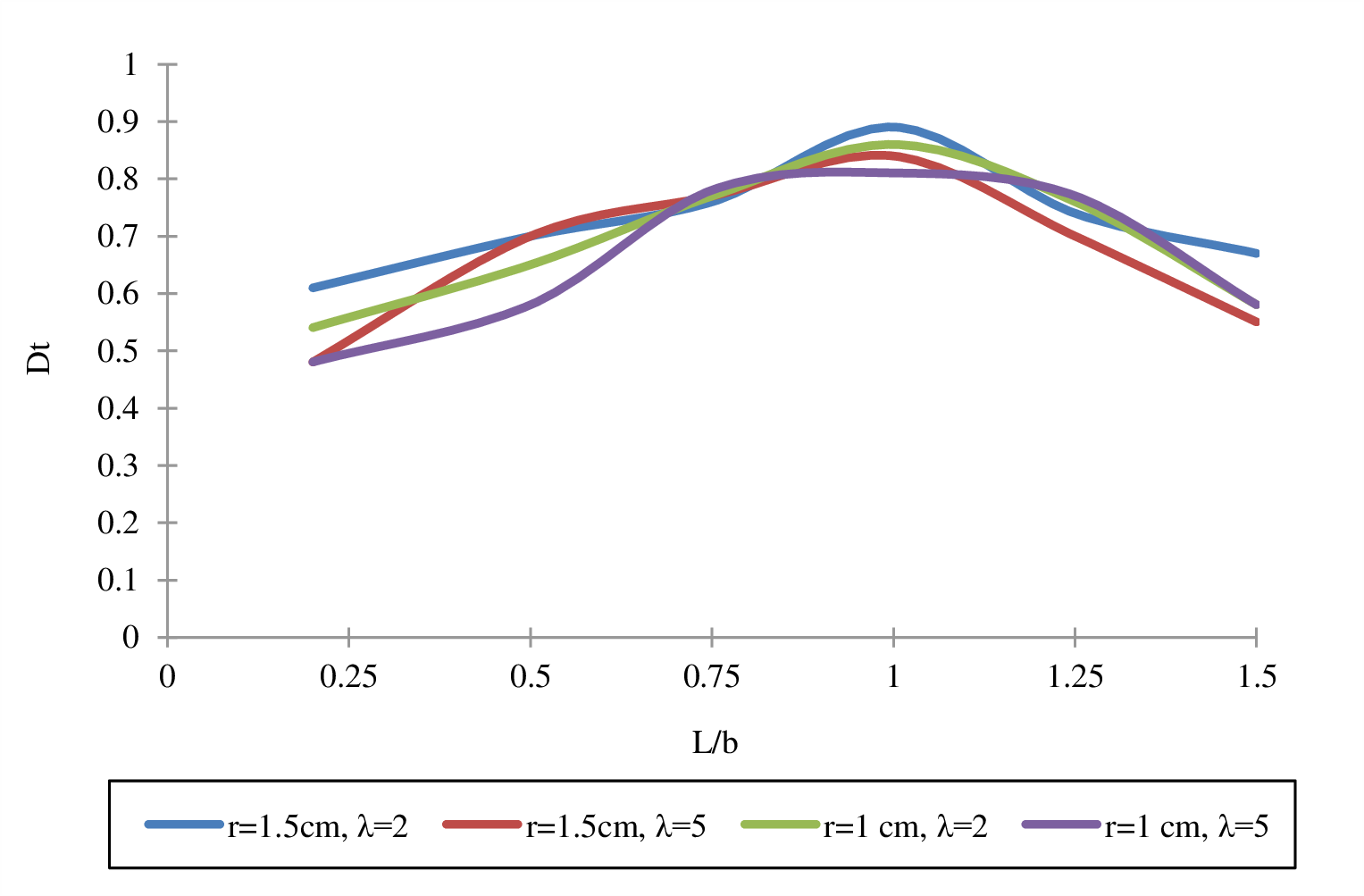

Supplement: S5 Fig — The vertical axis shows the mean fraction of dead cells in the tumor and the longitudinal axis shows the ratio of the distance between slots to the tumor radius. Allowable side effects are considered 5% in the healthy tissue. In all cases, the best treatment result achieved at Lb=1. (TIF) [file pone.0233219.s005.tif]
