# Supplementary material for: Use of microwave ablation for thermal treatment of solid tumors with different shapes and sizes—A computational approach
Source: PLoS One. 2020 Jun 15;15(6):e0233219. doi: 10.1371/journal.pone.0233219 (PMC7295236; doi:10.1371/journal.pone.0233219)
Supplement: S1 Text — (DOCX) [file pone.0233219.s006.docx]

**Supplementary Information**

# Mesh independency

To check the grid independency and identify the proper number of the required elements, multiple versions of the computational mesh are created. An appropriate cell number is chosen as a trade-off between the computing resources cost and the results of modeling [1]. Fig S1 demonstrates the relationship between the temperature and the number of required elements at a critically sensitive point, 2.5 mm away from the slot center at the frequency of 2.45 GHz for prolate tumor with λ=2. Case 4 and 5 almost have the same temperature, and thus, case 4 is the best choice because of lower computing costs. Results lead to 94,830 elements, where the solution is independent of mesh density. This assessment was done for other shapes of the tumor (spherical, oblate (λ=2 and λ=5), and prolate (λ=5)), and in each case, the optimal number of meshes were selected.

Water evaporation process

The power used for evaporation is introduced as a function of time [2], as follows:

| $Q_{E}=-\alpha\frac{dW}{dt}$ | (S1) |
| --- | --- |

where $\alpha$ is the water latent heat constant, which is 2260 [kJ/kg]. *W* is the tissue water density, which is calculated from the following relation [2]:

| $W=0.778\times\left\{ \begin{aligned} 1-\exp\left( \frac{T-106}{3.42} \right)T<103℃ \\ 0.03713T^{3}-11.47T^{2}1182T-40582 \\ \exp\left( \frac{T-80}{34.37} \right) T>104℃ \end{aligned} \right. 103℃<T\leq104℃$ | (S1) |
| --- | --- |

# Cell death process during thermal ablation

Generally, with the approach of temperature to the critical temperature, the biological operations of the cells have been disordered, and the cell death process began [3]. Cell’s death is a time-dependent process in which the rate of change in cell’s fraction is a function of temperature. In previous studies on MWA, the single temperature threshold is used to declare cell condition, above which the cells are instantaneously considered dead, and below it, the cells stay alive [4, 5]. They take no account of the treatment duration and level of temperature, even thoughit is known that this factor has an important effect on the cell’s response to the temperature gradient. The conversion of the living cells to the damaged and dead ones is needed to meet the cytotoxic temperature. After initiation of cell’s death process, a period of time is needed for all of the cells to be killed.

Fig S2 shows the state of the cells located at the radius of the tumor during the treatment. Although, after 8 minutes, the required temperature for cell death is provided, depending on the percentage of living cells, 17 minutes is needed to eliminate the tumor. The percentage of the damaged cells increases over a period of time, while as the treatment continues, all of them disappear. Therefore, the dimensions of the ablation zone are not determined by the boundary temperature.

## The effect of tumor size on treatment

Fig S3(A) shows the mean percentage of dead cells inside the spherical tumor. To study the side effects in normal tissue around the tumor, a point to the extent of 2.5 mm from the tumor wall located in the centerline has been examined. Results demonstrate that to eliminate more cancerous cells, a longer time is required. For tumor size of 0.5 cm, the treatment of the whole tumor with the fewest side effects (approximately 5%) is possible. But with an increase in tumor size, this value increases to 30% and 45% in tumors with a radius of 1cm and 1.5 cm, respectively. As this amount of side effects may cause termination of an organ containing the tumor, ablation of the whole tumor is not possible in larger ones. Cell death fractions up to 10%at 2.5 mm away from the radius of the tumor have been considered as an acceptable side effect. In general, the side effect is a limiting factor for hyperthermia. In the allowable range of side effects that are considered, 92% of cancer cells died in 4.5 minutes for the smallest tumor. The optimum treatment times for 1 cm and 1.5 cm radius tumor are respectively 13 min and 16 min, while the average percentage of dead cells reached 85% and 76%, respectively. Hence, with an increase in the tumor radius, the volume of the ablation zone decreases. Fig S3(C) demonstrates another critical feature of the cell death model: the fraction of the vulnerable cells. At the optimum treatment time discussed above, vulnerable cells comprise 25%, 23%, and 20% of the total cells in 0.5 cm, 1 cm, and 1.5 cm radius tumors, respectively. These fractions are higher than the dead cells around the tumor. This result further clarifies the importance of accounting damaged cells.

# Evaluation of treatment efficacy in different tumor shapes and sizes

The effect of the volume of tumors in various shapes is illustrated in Fig S4. The duration time of treatment depends on the amount of allowable side effects. The maximum volume of tumors that can be ablated at the allowable side effect is a 1% decrease for every 1 cm^3^ increase in volume. The optimum ablation time achieved at a constant allowable side effect in prolate shape remains constant in various volumes, but in oblate tumors, a lower aspect ratio has the highest duration time.

# Optimization slots distance according to the tumor shape and size

Fig. S5 indicates the average percentage of dead cells inside the tumor for various aspect ratios (L/b) in different tumor sizes with constant side effects. The allowable average of dead cells is assumed 5% in the surrounding area. In various *L*/*b*, the mean fraction of dead cells in the tumor is achieved at the optimum treatment time, in which the side effects have the minimum value. Results show that *L*/*b*=1 leads to the best treatment outcome. Using the optimized double slot antenna may destroy a 23% amount of tumor while it does not increase the side effects. As a result, shape and size of a tumor should be considered in order to optimize different parameters such as the position of the antenna in the patient’s body. As a result, in this study, it is suggested to use different antennas to treat tumors in various shapes and sizes.

**References**

1. Moradi Kashkooli F, Soltani M, Rezaeian M, Taatizadeh E, Hamedi M-H. Image-based spatio-temporal model of drug delivery in a heterogeneous vasculature of a solid tumor — Computational approach. Microvascular Research. 2019;123:111-24. doi: <https://doi.org/10.1016/j.mvr.2019.01.005>.

2. Yang D, Converse MC, Mahvi DM, Webster JG. Expanding the Bioheat Equation to Include Tissue Internal Water Evaporation During Heating. IEEE Transactions on Biomedical Engineering. 2007;54(8):1382-8. doi: 10.1109/TBME.2007.890740.

3. He X, Wolkers WF, Crowe JH, Swanlund DJ, Bischof JC. In situ thermal denaturation of proteins in dunning AT-1 prostate cancer cells: implication for hyperthermic cell injury. Annals of biomedical engineering. 2004;32(10):1384-98. Epub 2004/11/13. PubMed PMID: 15535056.

4. Saccomandi P, Schena E, Massaroni C, Fong Y, Grasso RF, Giurazza F, et al. Temperature monitoring during microwave ablation in ex vivo porcine livers. European journal of surgical oncology : the journal of the European Society of Surgical Oncology and the British Association of Surgical Oncology. 2015;41(12):1699-705. Epub 2015/10/05. doi: 10.1016/j.ejso.2015.08.171. PubMed PMID: 26433708; PubMed Central PMCID: PMCPMC5513178.

5. Wu X, Liu B, Xu B. Theoretical evaluation of high frequency microwave ablation applied in cancer therapy. Applied Thermal Engineering. 2016;107:501-7. doi: <https://doi.org/10.1016/j.applthermaleng.2016.07.010>.
